# Metastatic cardiac tumor from urothelial carcinoma detected by transthoracic echocardiography: a case report

**DOI:** 10.1186/s13256-015-0740-3

**Published:** 2015-11-16

**Authors:** Yasuteru Nakashima, Katsutoshi Tanioka, Toru Kubo, Naohito Yamasaki, Ichiro Yamasaki, Taro Syuin, Hiroaki Kitaoka

**Affiliations:** 1 Department of Cardiology, Neurology and Aging Science, Kochi Medical School, Kochi University, Oko-cho, Nankoku-city, Kochi 783-8505 Japan; 2 Department of Urology, Kochi Medical School, Kochi University, Oko-cho, Nankoku-city, Kochi 783-8505 Japan

**Keywords:** Cardiac metastasis, Echocardiography, Urothelial carcinoma

## Abstract

**Introduction:**

Cardiac metastasis of urothelial carcinoma is a very rare but clinically important complication. Most cardiac metastases are asymptomatic; symptoms from cardiac metastasis were seen in advanced stage and many of these cases were reported to have a poor prognosis. So it is important to find asymptomatic cardiac metastasis and to start chemotherapy early in order to improve the patient’s prognosis.

**Case presentation:**

A 73-year-old Asian man was referred to our hospital because of a right ventricular tumor. He had a history of left ureteral cancer 9 years ago. In screening echocardiography for paroxysmal atrial fibrillation, a low echogenic tumor was detected in his right ventricular apex, and characteristic ST segment elevation was detected in electrocardiography. An ^18^F-fluorodeoxyglucose positron emission tomography revealed abnormal uptake in his right ventricular apex tumor and prostate, and a biopsy of the prostatic tumor showed urothelial carcinoma cells. He received systemic gemcitabine, paclitaxel and cisplatin chemotherapy for the urothelial carcinoma, and the cardiac tumor size was reduced temporarily. Finally, he died of multiple organ failure 16 months after his first admission, but his survival period was relatively longer than previous reports.

**Conclusions:**

We experienced a case of a metastatic cardiac tumor from urothelial carcinoma. We found asymptomatic cardiac metastasis by screening echocardiography and electrocardiography. Our patient received systemic chemotherapy and his survival period was relatively longer than previous reports. Electrocardiography and echocardiography may be useful to find asymptomatic cardiac metastasis of neoplasms.

## Introduction

Cardiac metastasis from urothelial carcinoma is very uncommon. To the best of our knowledge, only a small number of cases were reported and most of the cases had poor prognosis. We present a case of a metastatic cardiac tumor from urothelial cell carcinoma that was incidentally detected by screening echocardiography. We treated the patient with systemic chemotherapy and succeeded in temporarily reducing the tumor size. The patient had 16 months of survival from diagnosis of cardiac metastasis, which is a longer period than other cases reported in the literature.

## Case presentation

A 73-year-old Asian man was referred to our hospital because of a right ventricular tumor. He had a history of left ureteral cancer, and he had undergone nephroureterectomy of his left kidney 9 years ago and transurethral resection of a bladder tumor (TUR-Bt) for intravesical recurrence. After four histories of TUR-Bt, no evidence of cancer recurrence was found for a period of 6 years. However, echocardiography for cardiac screening of paroxysmal atrial fibrillation revealed a low echogenic tumor in his right ventricle (RV), and he was admitted to our hospital for further examination.

**Fig. 1:**
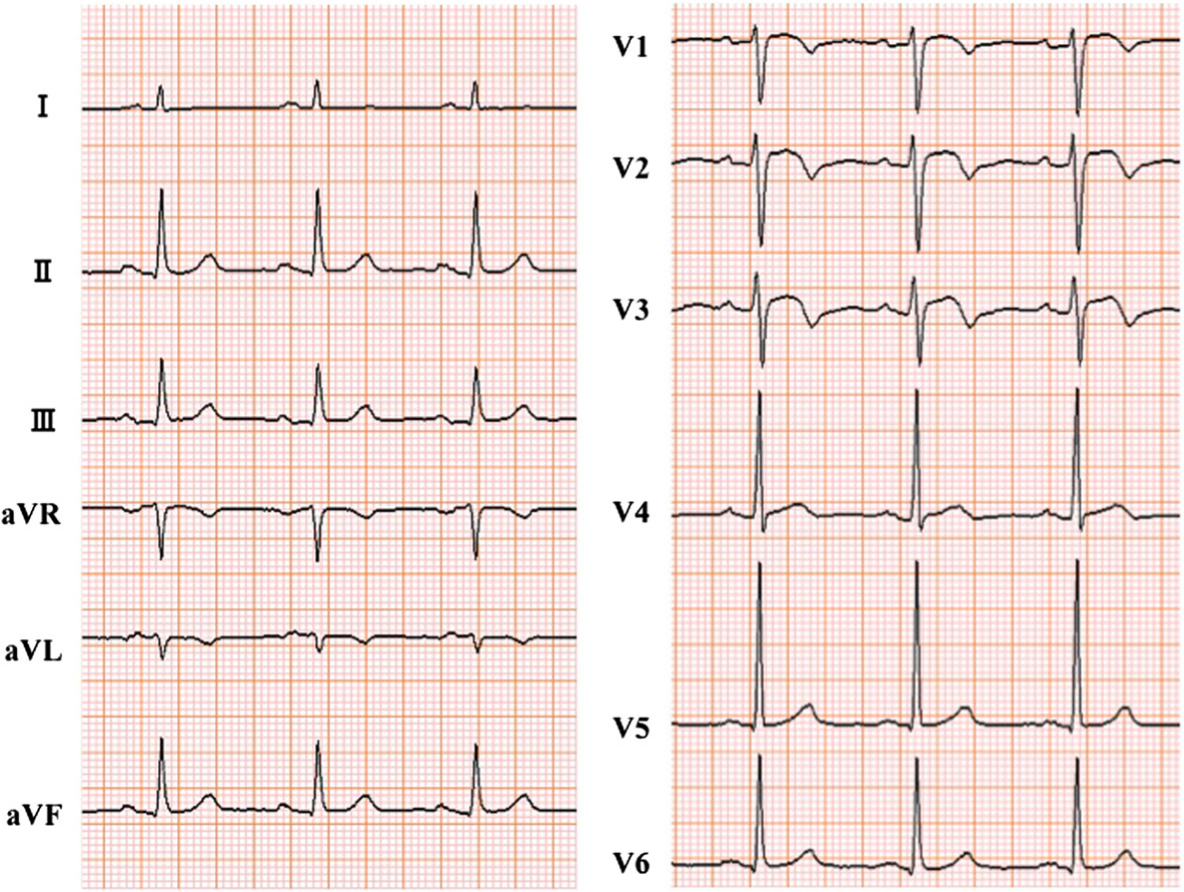
Electrocardiography at first admission for cardiac evaluation. Heart rate was 75/minute with normal axis. However, mild ST elevation and T inversion were detected in V1–3

**Fig. 2:**
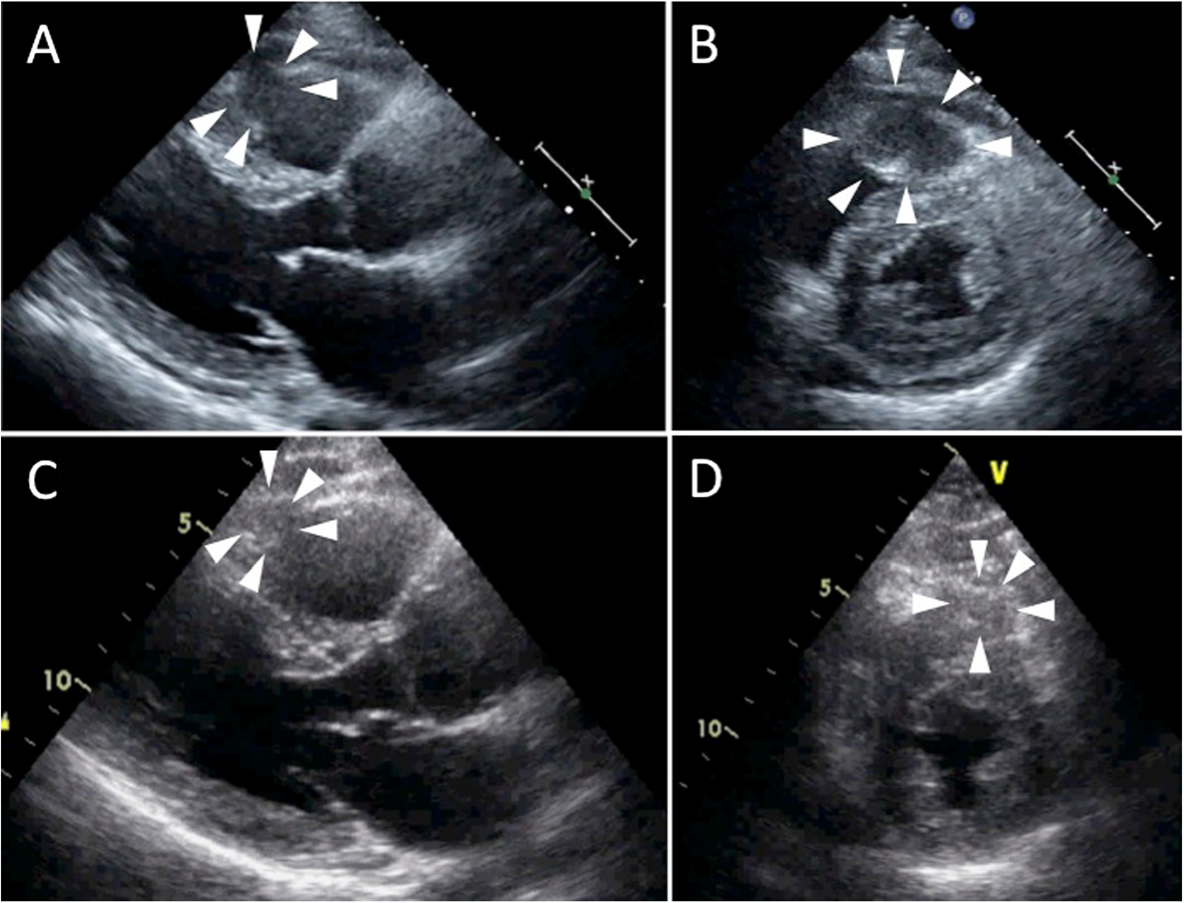
Transthoracic echocardiography. **a**, **b** Echocardiography before chemotherapy (a, diastolic phase; b, systolic phase): a low echogenic tumor was detected in the right ventricle apex wall (*white arrowheads*). **c**, **d** Echocardiography after two courses of chemotherapy (c, diastolic phase; d, systolic phase): right ventricle apex tumor had become smaller *(white arrowheads)*

The results of a physical examination were almost within normal limits, and laboratory tests showed mild chronic kidney disease (creatinine level of 1.22 mg/dl and blood urea nitrogen level of 21 mg/dl) and a mildly elevated brain natriuretic peptide level (178 pg/ml). In electrocardiography (ECG), mild ST-segment elevation and T wave inversion were revealed in V1–3 (Fig. 1). Transthoracic echocardiography revealed a 35×35 mm low echogenic tumor that was invading the myocardium of the apex of his RV, and the border of the tumor was poorly defined. There was a small amount of pericardial effusion and no detectable valvular abnormality (Fig. 2a, b).

**Fig. 3:**
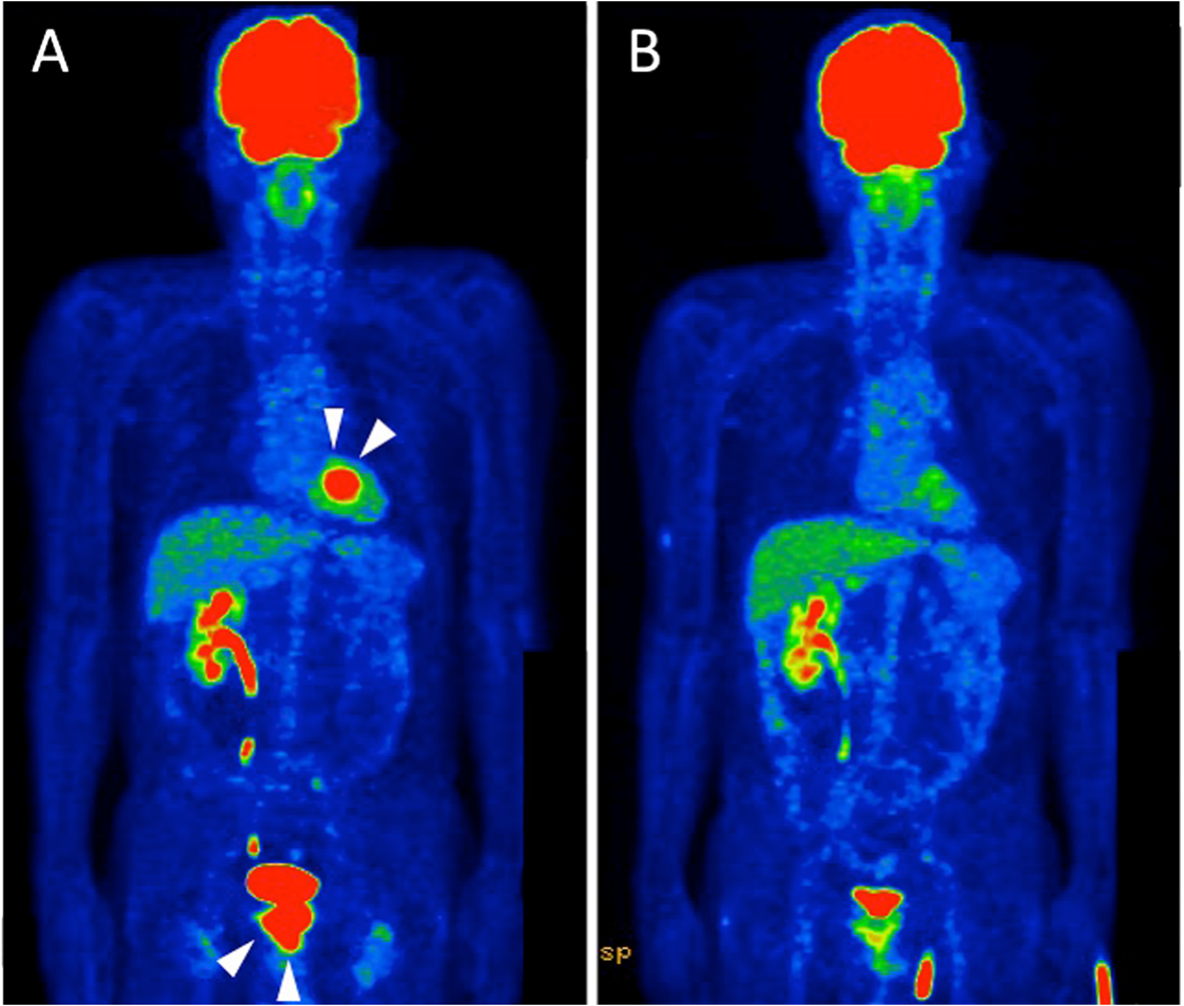
The patient’s ^18^F-fluorodeoxyglucose positron emission tomography. **a** His ^18^F-fluorodeoxyglucose positron emission tomography before chemotherapy: abnormal uptake was detected in the right ventricle apex tumor and prostate tumor (*white arrowheads*). **b** His ^18^F-fluorodeoxyglucose positron emission tomography after chemotherapy: uptake in the right ventricle apex tumor and prostate tumor was reduced

We suspected the tumor to be a metastasis from urothelial cancer based on its echocardiographical features and the patient’s past history. We tried to detect the primary lesion by ^18^F-fluorodeoxyglucose positron emission tomography (FDG-PET), and it showed abnormal FDG uptake in his RV apex tumor and prostate (Fig. 3a). Pelvis magnetic resonance imaging (MRI) revealed another lesion invading his prostate, and a biopsy of the prostate lesion showed carcinoma cells suspected to be from the urothelial carcinoma with squamous differentiation. Although the tumor was localized on his RV apical wall, and the risk of RV apical wall perforation due to cardiac biopsy was therefore considered to be relatively high, we recommended surgical or transvenous biopsy of the RV tumor for definite diagnosis of a metastatic cardiac tumor. However, he did not wish to undergo a biopsy.

We decided to perform systemic chemotherapy for the urothelial carcinoma and cardiac lesion. The patient received gemcitabine, paclitaxel and cisplatin therapy (GTC) for the urothelial carcinoma. After two courses of GTC therapy (75 and 50 % dose), the tumors of the prostate and RV apex wall became smaller, and FDG uptake of the tumors was also reduced as shown by follow-up FDG-PET (Figs. 2c, d and 3b). We suspected the RV tumor to be a metastatic cardiac tumor from urothelial carcinoma without pathological analysis because chemotherapy for the urothelial carcinoma was also effective for the RV tumor.

However, a new pubic bone metastasis was also revealed by follow-up FDG-PET. Radiation therapy was then performed for pubic bone metastasis (3 Gy×18 times, total 54 Gy). After the radiation therapy, chemotherapy was continued but the regime was changed to gemcitabine and cisplatin (GC) because of renal dysfunction. However, after three cycles of GC chemotherapy (50 % dose each), lung metastasis and adrenal gland metastasis were also detected, and chemotherapy itself was discontinued because of renal dysfunction, bone marrow suppression, and poor performance status.

Finally, he died from multiple organ failure 16 months after his first admission. An autopsy was not performed because permission could not be obtained from his family.

## Discussion

According to a recent review, cardiac metastasis from a malignant neoplasm is not rare. In past autopsy studies, the incidences of cardiac metastasis were estimated to range from 1.7 to 14 % in patients with cancer and from 0.7 to 3.5 % in the general population [1]. By contrast, the incidence of primary cardiac malignant neoplasm ranged from only 0.001 to 0.28 % [2]. In addition, the incidence of cardiac metastasis is expected to increase because of the improvement of prognosis for patients with cancer that is associated with advances in cancer therapeutic strategies [1].

Bussani *et al.* reported a large series of autopsy cases in 2007 [2]. They examined the cases of 18,751 in-hospital deceased patients, and they found one or more malignant neoplasms in 7289 patients. They also found cardiac metastasis in 662 of the patients (9.1 % of all), and the most common cancers among cardiac metastases were lung cancer (39.2 % of cardiac metastasis cases from lung cancer), breast cancer (10.0 %), mesothelioma (9.4 %) and lymphoma/leukemia (10.0 %). It was also shown that mesothelioma, melanoma and lung cancer have a relatively high potential for cardiac metastasis [2].

By contrast, cardiac metastasis from urothelial carcinoma is very rare. According to the autopsy study from Bussani *et al.* only 12 of 307 patients with urothelial carcinoma had cardiac metastasis [2]. In fact, to the best of our knowledge, only a small number of cases of symptomatic cardiac metastasis from urothelial carcinoma have been reported in the English literature. The reason for the rarity of cardiac metastasis from urothelial carcinoma is unclear. However, it may be due to the metastatic pathway of urothelial carcinoma. Malignant tumors metastasize to the heart by four alternative pathways: direct extension, hematogenous spread, lymphatic spread, and intracavitary extension from the inferior vena cava [3]. In epithelial malignancies, including urothelial carcinoma, distal metastasis occurred mainly by the lymphatic pathway [3]. However, in the heart, lymphatic flow is directly from the endocardium to the epicardium, and lymphatics drain from the heart to the mediastinum. It is therefore speculated that tumor cells cannot easily reach the heart without lymphatic flow stagnating due to tumor emboli [2]. This mechanism of the lymphatic system may play an important role in the rarity of cardiac metastasis from urothelial carcinoma.

The prognosis of cardiac metastasis from urothelial carcinoma is poor. Hattori *et al.* showed in a review of 14 cases that most patients died shortly after the original diagnosis or first visit [4]. Systemic chemotherapy or surgical resection of the tumor resulted in a prognosis of relatively long survival in only a few patients [5–8]. In our case, cardiac metastasis was detected by screening echocardiography for paroxysmal atrial fibrillation before the patient had any complaint, and systemic chemotherapy for urothelial carcinoma resulted in relatively long survival. Thus, early detection of cardiac metastasis and prostatic recurrence of carcinoma may have been important for the relatively long survival of our patient.

In addition, in our case, ECG showed characteristic abnormality (mild ST elevation and T inversion in V1–3). The exact cause of this ECG abnormality is unknown, but myocardial injury of the RV apex wall attributed to the metastatic tumor may have resulted in the ECG abnormality. A similar ECG abnormality was reported by Na *et al.* in a case of mimicking ST-segment elevation in myocardial infarction [9]. This case suggested that new onset ECG abnormality might provide a clue for the diagnosis of cardiac metastasis.

## Conclusions

We experienced a case of a metastatic cardiac tumor from urothelial carcinoma. He received systemic chemotherapy and his survival period was relatively longer than previous reports. In metastasis to the heart, most patients are asymptomatic, and it is therefore important to detect ECG or echocardiographical abnormality, recognize cardiac metastasis, and start appropriate treatment as early as possible.

## Consent

Written informed consent was obtained from the patient for publication of this case report and accompanying images. A copy of the written consent is available for review by the Editor-in-Chief of this journal.
